# Expanding Access to Non-Medicalized Community-Based Rapid Testing to Men Who Have Sex with Men: An Urgent HIV Prevention Intervention (The ANRS-DRAG Study)

**DOI:** 10.1371/journal.pone.0061225

**Published:** 2013-04-16

**Authors:** Nicolas Lorente, Marie Preau, Chantal Vernay-Vaisse, Marion Mora, Jerome Blanche, Joanne Otis, Alain Passeron, Jean-Marie Le Gall, Philippe Dhotte, Maria Patrizia Carrieri, Marie Suzan-Monti, Bruno Spire, ANRS-DRAG Study Group

**Affiliations:** 1 Institut National de la Santé et de la Recherche Médicale U912 (SESSTIM), Marseille, France; 2 Aix Marseille Université, IRD, UMR-S912, Marseille, France; 3 Observatoire Régional de la Santé Provence Alpes Côte d’Azur, Marseille, France; 4 Groupe de Recherche en Psychologie Sociale, Université Lyon 2, Lyon, France; 5 Conseil Général des Bouches du Rhône, Marseille, France; 6 Université du Québec à Montréal, Chaire de recherche du Canada en éducation à la santé, Montréal, Québec; 7 Conseil Général des Alpes Maritimes, Nice, France; 8 Aides, Pantin, France; 9 CMS*-*Centre d’Information et de Dépistage Anonyme et Gratuit*-*Centre d’Information, de Dépistage et de Diagnostic des Infections Sexuellement Transmissibles du Figuier, Paris, France; Indiana University and Moi University, United States of America

## Abstract

**Background:**

Little is known about the public health benefits of community-based, non-medicalized rapid HIV testing offers (CBOffer) specifically targeting men who have sex with men (MSM), compared with the standard medicalized HIV testing offer (SMOffer) in France. This study aimed to verify whether such a CBOffer, implemented in voluntary counselling and testing centres, could improve access to less recently HIV-tested MSM who present a risk behaviour profile similar to or higher than MSM tested with the SMOffer.

**Method:**

This multisite study enrolled MSM attending voluntary counselling and testing centres’ during opening hours in the SMOffer. CBOffer enrolees voluntarily came to the centres outside of opening hours, following a communication campaign in gay venues. A self-administered questionnaire was used to investigate HIV testing history and sexual behaviours including inconsistent condom use and risk reduction behaviours (in particular, a score of “intentional avoidance” for various at-risk situations was calculated). A mixed logistic regression identified factors associated with access to the CBOffer.

**Results:**

Among the 330 participants, 64% attended the CBOffer. Percentages of inconsistent condom use in both offers were similar (51% CBOffer, 50% SMOffer). In multivariate analyses, those attending the CBOffer had only one or no test in the previous two years, had a lower intentional avoidance score, and met more casual partners in saunas and backrooms than SMOffer enrolees.

**Conclusion:**

This specific rapid CBOffer attracted MSM less recently HIV-tested, who presented similar inconsistent condom use rates to SMOffer enrolees but who exposed themselves more to HIV-associated risks. Increasing entry points for HIV testing using community and non-medicalized tests is a priority to reach MSM who are still excluded.

## Introduction

In France, men who have sex with men (MSM) represent the only group in which new HIV diagnoses have continued to increase since 2003, accounting for 40% of new infections in 2010 [1]. The most recent estimation of the size of the hidden French epidemic showed that approximately 28 800 individuals were HIV positive without knowing it, of whom an estimated 31% were MSM [2].

Currently, HIV testing is considered a fundamental prevention intervention for controlling the HIV epidemic [3], [4]. Indeed, a recent study in the United States estimated that in the general population, people unaware of their infections accounted for almost half of new HIV transmissions [5]. In France, almost 20% of newly HIV-tested MSM in 2010 were diagnosed late, i.e. they had a CD4 count lower than 200 cells/mm^3^ [1].

Despite both American and French Health authorities’ primary recommendation that routine HIV testing in general health care settings be implemented [6], [7], [8], [9], a recent study conducted in emergency departments of the metropolitan Paris area using “non-targeted” rapid tests identified very few new HIV diagnoses. Furthermore these positive diagnoses were often in people at late stages of infection, and most were among well-known high-risk groups [10]. This result suggests that the non-targeted screening strategy currently used throughout France is ineffective, and highlights the need to specifically target most-at-risk populations, including MSM.

In vulnerable groups such as MSM, it is also recommended to diversify testing offers to encourage those still unaware of their HIV status to go for testing [11]. In France, the only way to benefit from free and anonymous testing for HIV, hepatitis and STIs is through voluntary counselling and testing (VCT) centres, and until recently, HIV testing could only be performed by qualified medical staff. One good alternative to encourage less recently HIV-tested MSM to go for testing is community-based non-medicalized testing using HIV rapid tests [11].

The ANRS-ComTest study, a rapid HIV testing offer recently conducted in and carried out by a French community-based organisation, showed that this non-medicalized testing offer was able to reach MSM with high-risk sexual behaviours, a substantial proportion of whom had not been recently tested for HIV [12]. However, no comparative data with MSM tested with the medicalized HIV testing offer were provided in that study.

The present study aimed to evaluate another community-based, non-medicalized HIV testing offer (CBOffer) implemented in VCT centres outside of opening hours in parallel with the standard medicalized HIV testing offer (SMOffer) provided during opening hours. The objective was to verify whether the CBOffer could attract less recently tested MSM who had a risk behaviour profile similar to or higher than those attending the SMOffer.

## Methods

### Study Design, Recruitment and Main Outcome

This cross-sectional study was conducted from March 2010 to April 2011 in free and anonymous VCT centres based in three French cities: two in Paris, one in Marseille and one in Nice. These centres are usually open from Monday to Friday during standard working hours.

The study was designed to compare two populations attending two different HIV testing offers:

1. The community-based, non-medicalized HIV testing offer (CBOffer) was provided by community members from the French NGO *AIDES,* a community-based organization that works with HIV-exposed populations in outreach and prevention, in particular MSM. Although they are not healthcare workers, *AIDES* staff members performed the full testing procedure, and provided specific counselling, based on the motivational interview method which they were trained in [13]. They implemented non-medicalized HIV rapid testing (using VIKIA® HIV1/2, BioMérieux, France), on a dedicated evening once a week (Monday to Friday), outside of the various VCT centres’ opening hours. Participants voluntarily came for testing following a communication campaign in gay venues.
2. The standard medicalized HIV testing offer (SMOffer) also operated in the VCT centres’, but only on one day a week (Monday to Friday) during opening hours. Participants were recruited from among the centres’ male attendees who reported that they had sex with other men. The VCT centres’ staff carried out HIV testing using ELISA and Western blot tests.

Accessing the CBOffer (as opposed to accessing the SMOffer) is the outcome variable of the present analysis.

### Ethics Statement

This study was approved by the French Comité de Protection des Personnes Sud-Est III (2009-029B). All participants had to provide written informed consent before enrolment, and blood samples were collected from all participants at the end of their tests in order to avoid inaccurate diagnoses. The study was anonymous.

### Data Collection and Explanatory Variables

Immediately after their agreement to participate, and before being tested, all participants had to fill in a self-administered questionnaire. This took approximately one hour. The questionnaire gathered data about sociodemographic characteristics as well as information about the risk perception, HIV testing history and sexual behaviours in the previous six months.

The testing variable, based on the number of HIV tests carried out in the previous two years, was dichotomised into 0 or 1 versus >1.

One aspect of sexual risk reduction – risk avoidance – was assessed using an “intentional risk avoidance scale”. The items used to construct this score were adapted from a questionnaire from the OMEGA Cohort Study [14] but the score itself was specifically built for the present study. Eight four-point scale items (never = 1, rarely = 2, quite often = 3, very often = 4) (Table 1), were used to build a score of intentional risk avoidance in terms of eight defined at-risk situations, by computing the mean score for each participant for all eight situations. The higher the score, the more the participants avoided risk. The lower the score the more participants exposed themselves to “at-risk” situations. The Cronbach’s α calculated to assess reliability of this intentional risk avoidance scale was 0.74, which is considered as an acceptable level of reliability [15]. This scale was then dichotomised according to the median score of participants into: <2.5 and >2.5 (out of 4).

**Table 1:** Items used to compute the “intentional risk avoidance” score.

(1) I avoid anal intercourse with HIV positive partners, or partners whose HIV status I am not sure about
(2) I avoid meeting sexual partners in saunas, backrooms, adult video shops or outdoor gay venues
(3) I avoid one-night stands
(4) I avoid sex without a condom since it might lead to bleeding
(5) I try to restrict the number of partners with whom I have anal intercourse
(6) I avoid having anal intercourse when I have drunk too much or when I have taken drugs
(7) In a sauna, a backroom or in an outdoor gay venue, I restrict the number of partners with whom I have sexual contact
(8) I avoid attending saunas, backrooms, adult video shops and/or outdoor gay venues

Concerning their sexual practices, participants were invited to answer several questions about anal intercourse and condom use for three types of male partners (casual, regular and steady, as described elsewhere [16]) and about the HIV serostatus of these partners. Two major variables investigated at-risk sexual practices:

1. The inconsistent condom use (ICU) variable: participants who reported that they had not systematically used condoms during anal intercourse in the previous six months were classified as ICU. This variable was computed for each of the three possible types of male partner: steady, regular and casual.
2. The serosorting variable was also built for these three types of male partner. Among participants who reported having a sexual partner (of any type) in the previous six months, serosorting was defined as reporting ICU only with those partners the individual supposed or knew to be HIV negative. For example, it was considered that a participant engaged in serosorting in casual encounters when he reported ICU with HIV negative casual partners, but also reported using condoms systematically when he had anal intercourse with casual partners who were either HIV positive or of unknown serostatus.

In addition, the following question recorded participants’ perception of when their most recent risky sexual episode: “When, in your opinion, was your most recent sexual risk taking episode?”. Answers were dichotomised into <3 months and >3 months.

Data on type of housing was also gathered for the participants. Those who reported renting or owning their own accommodation - i.e. those reporting that they did not live with relatives or friends or in hotels etc. – were classified as having “stable housing”.

### Analyses

A mixed logistic regression model was built to determine factors associated with the fact of accessing either the CBOffer or the SMOffer (the outcome variable), taking into account the correlation between individuals within each VCT centre. Potential explanatory variables of this outcome variable were individually screened in univariate analyses and odds ratios were calculated for each of them. Variables with a significance level of ≤0.20 were considered eligible for inclusion in the multivariate model. A backward method based on the log-likelihood ratio test (entry threshold p-value ≤0.05) was then used to select factors independently associated with the outcome. Statistical analyses were performed using SPSS-17 software (SPSS, Inc., Chicago, Illinois, USA) and STATA 12 (StataCorp. 2011. *Stata Statistical Software: Release 12*. College Station, TX: StataCorp LP).

## Results

Participation in the study was offered to a total of 455 MSM. Of the 236 MSM (52%) who came for the CBOffer, 11% (25/236) subsequently refused to participate. Of the 219 (48%) MSM offered the SMOffer, 46% (100/219) subsequently refused to participate. In both offers, the main reason for refusal was a lack of time (48% and 59% of those who refused in the CBOffer and the SMOffer, respectively). Therefore the study group (n = 330) comprised 211 MSM in the CBOffer, and 119 MSM in the SMOffer. Three men were tested HIV positive in each group (CBOffer: 3/211 = 1.4% versus SMOffer: 3/119 = 2.5%; Fisher test: p = 0.37).

Sixty-three per cent of the whole study group were recruited in Paris, 25% in Marseille and 12% in Nice. Median age was 31 years, 49% lived alone, 63% had completed secondary school and 78% were in active employment. The large majority defined themselves as homosexual (81%) and a significant proportion (15%) as bisexual. Major self-reported characteristics of participants and observed differences (whether significant or not) between both offers, are displayed in Table 2.

**Table 2:** Comparisons of the characteristics of men who have sex with men accessing the standard medicalized testing offer with those accessing the community-based non-medicalized testing offer (univariate analyses, n = 330).

Variables	Items	Whole sample (n = 330)	CBOffer (n = 211)	SMOffer (n = 119)	*p* *
		%	%	%	
***Demographics***					
Age	median [IQR]	31[25–39]	33[26–41]	26[23–35]	*<0.001*
Housing‡	Renter or owner	74.8	80.1	65.6	
	In a hotel, family etc.	23.0	17.5	32.8	*0.001*
Living alone‡	No	50.0	47.4	54.6	
	Yes	48.8	51.2	44.5	*0.12*
***HIV testing and risk behaviours***					
No. of HIV tests in the previous 2 years	None or 1	44.8	50.7	34.5	
	More than 1	54.8	49.3	64.7	*0.10*
Perceived most recent risk-taking episode‡‡	>3 months	33.0	55.9	66.4	
	≤3 months	59.7	35.1	29.4	*0.12*
Intentional risk avoidance score** ‡‡	<2.5 (out of 4)	46.1	50.2	38.7	
	≥2.5 (out of 4)	47.3	43.6	53.8	*0.02*
Sex under the influence of cannabis	No	77.3	80.6	71.4	
	Yes	22.7	19.4	28.6	*0.06*
***Sexual life*** ***					
Total no. of male sexual partners	median [IQR]	8 [3]–[18]	9 [3]–[18]	8 [4]–[15]	*0.10*
Having casual male partner(s) (**CMP**)	No	13.9	13.3	15.1	
	Yes	86.1	86.7	84.9	*ns*
No. of casual male partners CMP	median [IQR]	6 [2]–[13]	7 [2]–[15]	5 [2]–[11]	*0.13*
ICU with CMP‡	No	55.5	55.0	56.3	
	Yes	42.7	43.1	42.0	*ns*
Serosorting with CMP	No	91.2	90.0	93.3	
	Yes	8.8	10.0	6.7	*ns*
Having regular male partner(s) (**RMP**)	No	43.3	42.7	44.5	
	Yes	56.7	57.3	55.5	*ns*
No. of RMP	median [IQR]	1 [0–2]	1 [0–2]	1 [0–2]	*0.15*
ICU with RMP‡	No	74.2	74.9	73.1	
	Yes	24.5	24.2	25.2	*ns*
Serosorting with RMP	No	90.6	92.4	87.4	
	Yes	9.4	7.6	12.6	*0.08*
ICU with CMP and/or RMP‡	No	47.3	47.4	47.1	
	Yes	50.6	50.7	50.4	*ns*
Having steady male partner(s) (**SMP**)	No	56.7	60.7	49.6	
	Yes	43.3	39.3	50.4	*0.04*
No. of SMP	median [IQR]	0 [0–2]	0 [0–2]	1 [0–2]	*ns*
ICU with SMP	No	73.0	76.3	67.2	
	Yes	27.0	23.7	32.8	*0.009*
Serosorting with SMP	No	82.7	86.3	76.5	
	Yes	17.3	13.7	23.5	*0.02*
***Meeting places of casual male partners***					
Internet	No	40.0	42.6	35.3	
	Yes	60.0	57.4	64.7	*ns*
Saunas, backrooms, adult video shops	No	53.9	45.0	69.7	
	Yes	46.1	55.0	30.3	*<0.001*
Bars, discos	No	56.4	52.1	63.9	
	Yes	43.6	47.9	36.1	*0.06*
Workplace	No	92.1	94.3	88.2	
	Yes	7.9	5.7	11.8	*0.07*
Outdoor gay venues	No	77.9	74.9	83.2	
	Yes	22.1	25.1	16.8	*0.19*

**CBOffer**: community-based non-medicalized HIV testing offer; **SMOffer**: standard medicalized HIV testing offer; **IQR**: interquartile range; **ICU**: inconsistent condom use; **ns**: not significant (i.e. p-value ≥0.20).

* This column displays p-values for each variable tested in univariate logistic regression, and shows whether differences between the two subsamples are significant or not.

** The higher the score, the more participants were able to avoid risk; the lower the score the more participants exposed themselves to “at-risk” situations.

*** The “Sexual life” section variables were computed for the previous 6 months.

‡ Missing data accounted for <5%.

‡‡ Missing data accounted for <10%.

Univariate analyses showed that MSM who accessed the CBOffer were significantly more likely to report having had one or no HIV test in the previous two years, and to have an intentional risk avoidance score of less than 2.5/4 than participants enrolled in the SMOffer. Additionally, CBOffer attendees engaged less in serosorting in regular or steady encounters, and their most recent self-perceived risk-taking episode was significantly more recent (<3 months) than that reported by their SMOffer counterparts (Table 2).

Findings also showed that MSM who voluntarily came for testing to the CBOffer were older, more likely to rent or to own their accommodation and to live alone. On the whole, these men reported having more male sexual partners, meeting casual partners more often in saunas, backrooms or adult video shops with booths for having sex, in bars or discos, and in outdoor gay venues. However they were less likely to report having had a steady male partner in the previous six months than participants in the SMOffer (Table 2).

Interestingly, no differences were found between ICU levels in both testing offers, irrespective of the partner type: casual, regular, or steady (Table 2).

Participants in the CBOffer did not differ from their SMOffer counterparts with respect to the following demographic characteristics: having completed secondary school, being in active employment, the place of birth and how they defined their sexual identity. They were not significantly more often victims of verbal abuse (20% vs. 24%) or victims of physical abuse (2% vs. 3%), and levels of knowledge and acceptance of their sexual orientation by their relatives and friends were similar to those found for SMOffer participants. Indeed, the sexual orientation of all participants in both testing offers was well accepted on the whole by their family and friends: 40% by fathers, 53% by mothers, 56% by brothers and sisters, 48% by colleagues, and 67% by friends. Only a minority of participants reported rejection (from 0% to 7%, depending on the type of personal relationship).

Finally, after adjustment for age, independent factors associated with the fact of accessing the CBOffer were as follows: reporting to have had only one or no HIV tests in the preceding two years, having a lower sexual risk avoidance score, meeting casual partners in saunas, backrooms or adult video shops, and having stable housing (Table 3).

**Table 3:** Factors associated (OR) and independently associated (aOR) with accessing the community-based non-medicalized HIV testing offer (multivariate analyses controlling for all sites, p<0.05, n = 300).

Variables	CBOffer (n = 193)	SMOffer (n = 107)	OR [CI 95%]	aOR [CI 95%]
	%	%		
Age (median [IQR])	33 [26–40]	26 [23–36]	1.06 [1.03–1.09]	1.04 [1.00–1.07]
Living in a hotel, family etc.	18.1	31.8	1	1
Renter or owner of housing	81.9	68.2	2.56 [1.42–4.61]	2.40 [1.20–4.79]
HIV tests in the previous 2 years: >1	47.7	63.6	1	1
HIV tests in the previous 2 years: none or 1	52.3	34.4	1.85 [1.12–3.13]	2.27 [1.27–4.17]
Mean avoidance score <2.5 (out of 4)*	52.8	40.2	1	1
Mean avoidance score ≥2.5 (out of 4) *	47.2	59.8	0.54 [0.33–0.89]	0.38 [0.22–0.70]
No casual partners, or not met in saunas, backrooms, adult video shops	46.1	70.1	1	1
Casual partners met in saunas, backrooms etc.	54.9	29.9	2.75 [1.64–4.64]	2.62 [1.40–4.88]

**CBOffer**: community-based non-medicalized HIV testing offer; **SMOffer**: standard medicalized HIV testing offer; **OR**: odds ratio; **aOR**: adjusted odds ratio; **CI**: confidence interval; **IQR**: interquartile range.

* The higher the score, the more the participants were able to avoid risk; the lower the score the more participants exposed themselves to “at-risk” situations.

As the sexual risk avoidance score and serosorting with one’s steady partner were collinear, both variables could not be kept in the multivariate model. However, keeping the adjustment for age (aOR: adjusted odds ratio [95% CI: confidence interval] = 1.03[1.00–3.34]), serosorting with one’s steady partner was independently associated with accessing the CBOffer (aOR [95% CI] = 0.51[0.26–0.99]) when the avoidance score was removed from the model. The other factors of the first model (having had only one or no HIV tests in the preceding two years (aOR [95% CI] = 0.58[0.34–0.98]), meeting casual partners in saunas, backrooms or adult video shops (aOR [95% CI] = 1.91[1.09–3.34]), and having stable housing (aOR [95% CI] = 2.10[1.10–3.99])) all remained significantly associated with the outcome.

## Discussion

This is the first study comparing a completely non-medicalized HIV rapid testing offer conducted by community members with the standard medicalized HIV testing offer among MSM living in France.

The major finding is that the community-based non-medicalized HIV rapid testing offer did in fact attract MSM who were much less likely to have been tested during the two previous years than those attending the standard HIV testing offer. In addition, although the CBOffer attracted MSM with similar levels of ICU as their counterparts tested with the SMOffer, the former were at greater risk because they exposed themselves more to at-risk situations and because they attended saunas, backrooms or adult video shops more often.

These findings are consistent with two previously published articles about non-medicalized HIV testing offers, in France and in the UK, which showed that this kind of offer managed to reach MSM who reported both high levels of ICU and to have never been or not recently been tested [12], [17]. Unlike the UK study where the rapid testing program was staffed by professionals from genitourinary medicine clinics [17], our CBOffer was completely non-medicalized and staffed by community-based members. Furthermore, although the French testing program mentioned above was staffed by individuals working for a French community organization within their own premises [12], their study did not provide any data about the benefits of the non-medicalized offer over the standard one as, unlike in our study, there was no control group to make a comparison.

We recently conducted another study in French VCT centres which highlighted that MSM not recently tested were more likely to report difficulties using condoms in several at-risk situations and were also less likely to report sexual risk reduction behaviours than recently tested MSM [18].

In the present study, a higher proportion of refusals to participate (46%) was seen in the SMOffer compared with the CBOffer (11%). This difference in refusal rates is probably linked to the fact that potential participants first went to the VCT centres with the intention of having the standard HIV test. When proposed the SMOffer, they had to provide written informed consent before enrolment in the study, and had to fill in a long questionnaire. Basically, administering the SMOffer took a much longer amount of time than that usually required when individuals go for standard testing to a VCT centre (indeed a lack of time was the principal reason for subsequently refusing to participate: 59%). We must therefore consider the fact that we probably selected quite a specific population of MSM in the SMOffer. Indeed, in our previous study in VCT centres, the ICU level with partners other than steady partners was 44% [18], whereas in the present study it was 51% among those recruited in the SMOffer.

The absence of any difference in ICU levels between participants of both the CBOffer and SMOffer might be partially explained by the fact that the selected population in the SMOffer did not correspond to attendees who usually come for testing in the participating VCT centres (cf. our previous study [18]). Indeed, SMOffer attendees were at higher risk, and probably felt more concerned by HIV issues and prevention research than these regular VCT centre attendees. Although this selection bias minimizes the potential appeal of community testing to high risk MSM it does not weaken the results of the study.

The score of intentional risk avoidance we built in these analyses had an acceptable reliability level (Cronbach’s α = 0.74 [15]) and was able to capture subtleties regarding HIV risk behaviours, undetectable using criteria solely based on ICU. This score was lower among participants in the CBOffer, highlighting its attractiveness to a specific subpopulation of MSM more exposed to at-risk situations. Indeed, although serosorting with one’s steady partners was no longer significant in the final model due to collinearity with the intentional risk avoidance score, MSM who voluntarily came to the CBOffer were less likely to engage in serosorting in steady partnerships than their SMOffer counterparts. This is interesting when one considers that a US study recently showed that 68% of newly diagnosed HIV cases among MSM occur in such partnerships [19], and that other studies have shown that serosorting can decrease the risk of HIV seroconversion [20], [21]. Furthermore, MSM tested in the CBOffer were significantly more likely to meet their casual partners in saunas, backrooms or adult video shops. A French study conducted in these kinds of venues estimated that the HIV incidence in the MSM population which frequent such venues was higher (3.8% person-years) than that in the whole population of MSM (1% person-years) [22], [23]. The present study therefore suggests that MSM attracted by the community-based testing offer could be at greater risk of HIV infection than those attending the standard testing offer.

In the present study, MSM who voluntarily came to the CBOffer seemed to be more economically independent as they were significantly older and more likely to have stable housing than those attending the SMOffer. This is in sharp contrast to findings from the UK’s rapid community HIV testing program which attracted significantly younger MSM than those tested at genitourinary medicine clinics [17]. Nevertheless, in the present study, although they tended to have a more active gay life, in particular by going more often to recognized gay venues where HIV prevention is very active, the level of recent testing was significantly lower in participants in the CBOffer than in those attending the SMOffer, suggesting that standard medicalized HIV testing performed in VCT centres was not attractive to these MSM.

The first possible limitation of this study is that most of the participants (63%) were recruited in Paris. This choice was a methodological one as Paris and its suburbs account for the largest proportion of the HIV epidemic among MSM living in France [24]. Accordingly, this metropolitan area accounted for the largest part of the target population of our study. Second, the CBOffer was carried out on a specific evening every week, and this fact may also have influenced the recruitment process. However, it is unlikely that this possible bias impacted our results since the proportion of MSM in active employment did not differ between those enrolled in both testing offers. Furthermore, being in active employment was not found to be a confounder since this variable did not change the odds ratios of the other variables in the final model (data not shown). Third, it is not possible to determine which aspect of the intervention – opening hours versus dedicated evening, medical staff versus community members, rapid versus Elisa tests – attracted more CBOffer enrolees. This study did not aim to address this point, but focused on implementing a testing offer which could be employed in the future to complement the existing standard testing offer in VCT centres. Unlike the CBOffer, it is unlikely that these centres will be able to offer the specific counselling provided by community members or indeed be able to open during evening hours.

Our results suggest that the CBOffer staffed by trained community members is feasible. It managed to reach those MSM who were less likely to have been previously tested, those who reported high levels of inconsistent condom use and those who reported more sexual risk attitudes than their counterparts tested in the standard medicalized offer.

We strongly recommend the implementation of this rapid testing offer in addition to other testing methods – including medicalized or home-based testing – as they do in fact complement each other [25], [26]. All testing methods reach different subgroups of high-risk MSM living in France and help to increase self-knowledge of HIV serostatus, which is a major factor in reducing HIV transmission [27], [28]. This is a further argument for recommending targeted screening [10].

Recently, the French government decided to authorize the use of HIV rapid tests by non-medical staff [29]. Given the low score for intentional avoidance of at-risk situations which we found in the target population, we therefore recommend that any counselling provided to MSM during community-based HIV testing be centred on sexual risk reduction strategies. When a counsellor meets an MSM who reports engaging in unprotected sex, then depending on the latter’s sex life and on the type of at-risk situations in which he finds himself, the counsellor should be able to propose concrete scenarios to help the MSM avoid risks.

Today many MSM are still excluded from HIV testing, especially in low and middle income countries where exposure to HIV risk is high, where MSM are highly stigmatized and therefore are less likely to go for standard testing. Increasing the number of entry points for non-medicalized HIV-testing by implementing community-based testing is a public health priority not only to control the HIV epidemic but also to reduce inequalities in access to HIV care.
