# Correction: Expanding Access to Non-Medicalized Community-Based Rapid Testing to Men Who Have Sex with Men: An Urgent HIV Prevention Intervention (The ANRS-DRAG Study)

**DOI:** 10.1371/annotation/73d8610b-c201-4889-889e-db0fba5a3ddc

**Published:** 2013-06-26

**Authors:** Nicolas Lorente, Marie Preau, Chantal Vernay-Vaisse, Marion Mora, Jerome Blanche, Joanne Otis, Alain Passeron, Jean-Marie Le Gall, Philippe Dhotte, Maria Patrizia Carrieri, Marie Suzan-Monti, Bruno Spire, ANRS-DRAG Study Group

An affiliation of the last author was incorrect. 

Bruno Spire is correctly indicated as affiliated with institutions 1, 2 and 3, but is also affiliated with institution 8, Aides, Pantin, France, not with institution 7. 

In addition, there was an error in affiliation institution 1 for authors Nicolas Lorente, Marie Preau, Marion Mora, Jerome Blanche, Maria Patrizia Carrieri, Marie Suzan-Monti and Bruno Spire. 

Institution 1 should be: INSERM UMR912 (SESSTIM), Marseille, France. 

